# Test de diagnóstico rápido en la patología dermatológica pediátrica

**DOI:** 10.1016/j.aprim.2024.103061

**Published:** 2024-07-30

**Authors:** Luis Ortiz-González, Francisco Peral-Rubio, Basilio Narváez-Moreno

**Affiliations:** Departamento de Ciencias Biomédicas. Facultad de Medicina y Ciencias de la Salud. Universidad de Extremadura, Badajoz, España

Los test de diagnóstico rápido (TDR) son pruebas diagnósticas que están diseñadas para realizarse en la propia consulta, por el facultativo o por el personal auxiliar debidamente formado y sin la ayuda de un laboratorio, y que permiten obtener el resultado en el mismo acto asistencial en un periodo de tiempo comprendido entre 5 y 15 minutos. Deben tener una sensibilidad y especificidad adecuadas y un coste razonable para que puedan ser implementadas en la práctica clínica ordinaria. La técnica de recogida y procesamiento de las muestras debe ser sencilla y lo menos invasiva y molesta para el paciente^1^. Desde la reciente pandemia de la COVID-19, tanto la comunidad asistencial como el público en general han tomado conciencia de la importancia de la utilización de los TDR en la atención primaria de salud. Ello no solo es relevante para realizar un diagnóstico correcto de los diferentes procesos infecciosos, sino también para implementar las correspondientes medidas de aislamiento temporales adecuadas con objeto de evitar el contagio en la colectividad^1^.

En dermatología, los TDR son escasamente utilizados; en unos casos por su falta de disponibilidad, debido a que su uso inhabitual y su fecha de caducidad (hasta unos 18 meses después de su adquisición) no la justifiquen; y en otros casos, por el desconocimiento de su existencia y su utilidad. Además de la dermatoscopia y la ecografía cutánea, los TDR proporcionan al clínico un considerable salto cualitativo desde el punto de vista asistencial.

Presentamos dos casos clínicos en los que los TDR han permitido esclarecer la etiología del proceso e indicar el tratamiento adecuado.

El primero de ellos se trata de una niña, de ocho años y dos meses de edad, que presentó un cuadro de 48 horas de evolución caracterizado por fiebre (hasta 38,7 °C, axilar), tos productiva y lesiones cutáneas acrales levemente pruriginosas que aparecieron en las primeras 24 horas del inicio de la fiebre. En la exploración física, presentaba orofaringe hiperémica a nivel de pilares anteriores, sin exudados, y auscultación pulmonar sin hallazgos relevantes. A nivel cutáneo, encontramos, en manos, muñecas, tobillos y pies, pápulas y máculas eritematosas con afectación bilateral (fig. 1). Se realizó TDR inmunocromatográfico, que incluía reactivos para la detección de SARS-CoV-2, influenza A y B, adenovirus, virus respiratorio sincitial (VRS) y *Mycoplasma pneumoniae*, con resultado positivo para el virus influenza A. Se concluyó el diagnóstico clínico de gripe A asociado a exantema viral concomitante y se indicó tratamiento sintomático, con evolución favorable del proceso y resolución de la lesiones cutáneas a los siete días del inicio de las mismas^1^, ^2^, ^3^.

**Figura 1:**
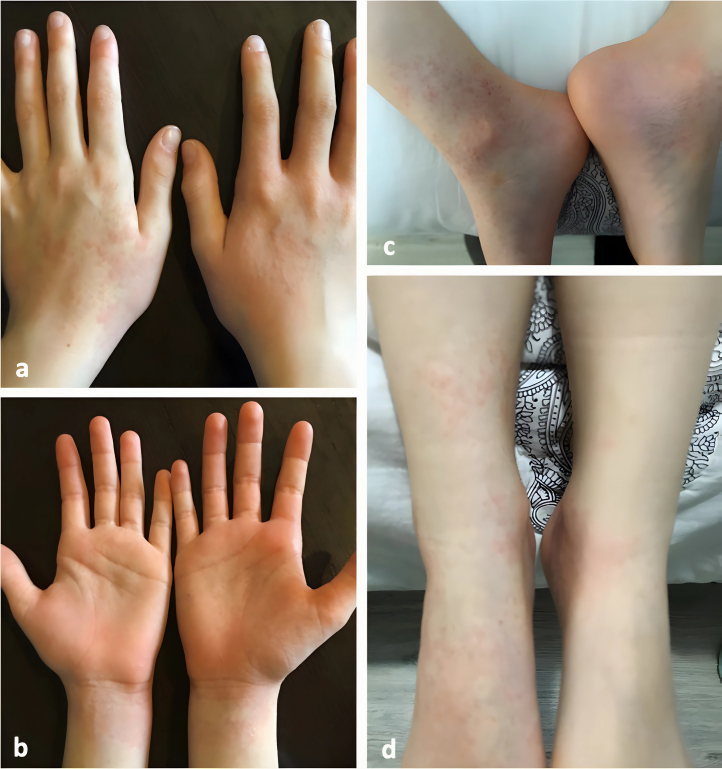
Máculas eritematosas en manos y muñecas (a, b), y en tobillos y pies (c, d).

El segundo caso corresponde a un niño de seis años de edad que presentó un exantema de 10 días de evolución localizado en el área perioral. Fue tratado con ozenoxacino tópico sin respuesta. En la exploración, presentaba elementos costrosos de coloración marronácea de distribución perioral (fig. 2). Ante la sospecha clínica de un cuadro de impétigo, se realizó TDR específico para estreptococo β-hemolítico del grupo A en muestra de exudado de las lesiones, con resultado positivo, por lo que se indicó antibioterapia oral dirigida consistente en fenoximetilpenicilina oral durante 10 días, además de tratamiento tópico con mupirocina, con remisión completa del proceso^4^, ^5^, ^6^.

**Figura 2:**
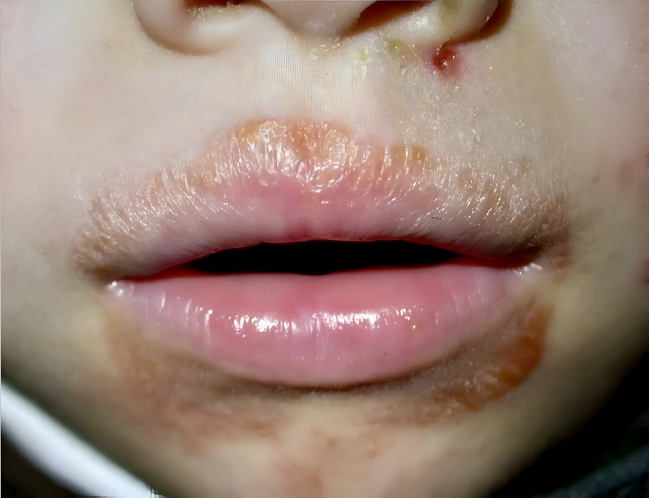
Impétigo perioral.

La sospecha clínica y los TDR permitieron hacer un diagnóstico etiológico en ambos pacientes y prescribir el tratamiento adecuado. El primero de ellos fue sintomático, y el segundo, específico para el agente implicado. En cualquier caso, la utilización de los TDR permite al clínico pasar del diagnóstico sindrómico, subjetivo y cargado de incertidumbre, al diagnóstico etiológico, disminuir el número de consultas sucesivas y disminuir la iatrogenia.

## Consideraciones éticas

Los autores confirman que se han obtenido todos los consentimientos requeridos por la legislación vigente para la publicación de cualquier dato personal o imágenes de pacientes, sujetos de investigación u otras personas que aparecen en los materiales enviados a Elsevier, se han realizado todos los procedimientos éticos y se han respetado los derechos de privacidad de los sujetos humanos.

Los autores conservan una copia escrita de todos los consentimientos y, en caso de que Elsevier lo solicite, aceptan proporcionar las copias o pruebas de que de dichos consentimientos han sido obtenidos.

## Financiación

Los autores manifiestan que no han recibido financiación alguna para la elaboración del manuscrito.

## Conflicto de intereses

Los autores declaran no tener ningún tipo de conflicto de intereses para la elaboración del manuscrito.
